# Synthesis of Graphene-Based Sensors and Application on Detecting SF_6_ Decomposing Products: A Review

**DOI:** 10.3390/s17020363

**Published:** 2017-02-13

**Authors:** Xiaoxing Zhang, Hao Cui, Yingang Gui

**Affiliations:** 1 State Key Laboratory of Power Transmission Equipment & System Security and New Technology, Chongqing University, Chongqing 400044, China; cuihaocqu@163.com (H.C.); yingang.gui@gmail.com (Y.G.); 2 School of Electrical Engineering, Wuhan University, Wuhan 430072, China

**Keywords:** graphene, sensors, synthesis, SF_6_ decomposing products

## Abstract

Graphene-based materials have aroused enormous focus on a wide range of engineering fields because of their unique structure. One of the most promising applications is gas adsorption and sensing. In electrical engineering, graphene-based sensors are also employed as detecting devices to estimate the operation status of gas insulated switchgear (GIS). This paper reviews the main synthesis methods of graphene, gas adsorption, and sensing mechanism of its based sensors, as well as their applications in detecting SF_6_ decomposing products, such as SO_2_, H_2_S, SO_2_F_2_, and SOF_2_, in GIS. Both theoretical and experimental researches on gas response of graphene-based sensors to these typical gases are summarized. Finally, the future research trend about graphene synthesis technique and relevant perspective are also given.

## 1. Introduction

The past several decades have witnessed increasing attention by researchers to carbon-based materials, especially those formed by sp^2^ C atoms such as carbon nanotubes and graphene owing to their unique structure, a soft membrane. The groundbreaking fabrication of graphene [1] has aroused considerable studies on their fundamental properties and applications. It has been experimentally proved [2] that graphene is only a one-atom thick layer of graphite, and in the meantime possesses a high Young’s modulus as well as superior thermal and electrical conductivities [3,4]. Given its high theoretical specific surface area of ~2600 m^2^/g, graphene displays a wonderful performance for surface chemistry [5,6,7]. These outstanding physical and chemical properties of graphene further contribute to its studies and applications in batteries, nanoelectronics, fuel cells, photovoltaics, catalysis, separation, and storage, along with gas sorption and gas sensing [8,9,10,11,12] as well. In terms of gas adsorption and sensing on graphene-based materials, a large amount of research [13,14,15,16,17,18] has been carried out for many years, aimed at exploiting new-fashioned types of graphene-based sensors that are capable of detecting individual gases with a relatively high sensitivity, which has already made great breakthroughs.

It has been well-known that sulfur hexafluoride (SF_6_) gas, a most commonly utilized kind of insulation gas in high voltage equipment of power systems, especially gas-insulated switchgear (GIS) due to its excellent chemically inert and wonder dielectric strength [19,20], is inevitably decomposed under partial discharge in a long running device. These decompositions further react with contaminants such as air or water vapor [21,22] and subsequently generate several components such as SO_2_, H_2_S, SO_2_F_2_, CF_4_ and SOF_2_ [23,24,25]. Referring to previous studies, they have been demonstrated as able to accelerate the facility corrosion rate and to increase system paralysis probability [26,27]. Consequently, it is of great significance and necessity to perform on-line detection of these typical gases of SF_6_ in order to estimate the operation status of GIS, guarding against more serious insulated faults.

Given the rapid development of graphene-based gas sensors and the essentiality of guaranteeing the normal running of GIS, more and more researchers are contributing by introducing novel graphene-based sensors for the sake of realizing the high-response detection of SF_6_ decomposition products. To more comprehensively understand such an application of graphene, this paper attempts to summarize what has hitherto been studied in the field. Section 2 describes the main synthesis techniques of graphene-based materials and their adsorption and sensing mechanisms. Section 3 illustrates the application of graphene-based sensors for detecting SF_6_ typical gases. Finally, Section 4 summarizes the research status of this field and puts forward the future developing trend of newfangled sensing materials, providing suggestions for further improvement in preparing graphene-based sensors applied in the field of electrical engineering.

## 2. Synthesis and Analysis of Graphene-Based Materials

### 2.1. Synthesis of Graphene-Based Materials

Since Konstantin [28] and Andre [1] first demonstrated the existence of graphene by a repeatable and successful synthesizing technique named micromechanical cleavage. A large number of researchers have been devoted to produce such an extraordinary substance in an effective and large-scale way.

Stankovich [29] prepared a material with comparable traits to those of graphene by reducing exfoliated graphene oxide sheets in water. Zhu et al. [30] proposed a facile, environmentally friendly, and possibly large-scale production approach to the preparation of graphene nanosheets, which is linked to the reduction of sugars with exfoliated graphite oxide (GO) as a precursor. Shukla et al. [31] reported a fast and inexpensive method to the synthesis of single- or few-layer graphene by cleaving off the bulk graphite on the substrate made of borosilicate glass or any insulating materials with ionic conductivity, which has promise for industrial level production for certain engineering applications. Martín-García et al. [32] showed that reduced GO functionalized zwitterionic surfactant can significantly improve the electrical conductivity of graphene film, thereby enhancing its quality. Obraztsov et al. [33] employed chemical vapor deposition (CVD) to synthesize graphene on a Ni substrate and found that a few graphene layers with thickness ranging from 1 to 2 nm grew successfully on the surface of the substrate, while the same approach failed to make the graphene adhere to a Si substrate, ascribed to the large lattice incompatibility between silicon and graphite. In another study by Obraztsov et al. [34], they employed a gas mixture of H_2_ and CH_4_ with a pressure ranging from 10 to 150 Torr and Si wafer, Ni, W, Mo, and some other metal sheets as substrates to synthesize graphene by direct current discharge plasma-enhanced chemical vapor deposition (PECVD). Results indicated that, although some twisted parts at the graphene do exist, the thick places account for the most proportions.

### 2.2. Adsorption and Sensing Mechanism of Graphene-Based Materials

For carbon materials, gas adsorption is primarily depended on physisorption on their surfaces, and sometimes the electrostatic and dispersion reactions that can be tuned by chemical substitution can happen as well. Two factors, namely the surface characteristic of the adsorbent and the properties of the target gas molecules, can influence the interaction strength and further determine the adsorbing rate [35]. Specifically, adsorbents that possesses a high specific surface area are good at adsorbing gas molecules with high polarizability but no polarity; those molecules with a high dipole moment can be effortlessly adsorbed on highly polarized surfaces. An absorbent with high electric field gradient surfaces is perfect for the adsorption of high quadrupole moment-targeted molecules [36]. Given that gas adsorption is mainly governed by physisorption, the graphene that has a large available surface area with high porosity can be an excellent candidate for applications in certain fields. Besides, graphene with nanoporous membranes has the potential to yield high selectivity through molecular size exclusion effects, since its high permeability based on a small thickness [37], which improves the practicability of graphene-based sensors. In 2007, Schedin et al. [18] studied the sensing performance of graphene towards NO_2_, NH_3_, H_2_O, and CO and proved that these gaseous chemicals can be detected individually, since graphene-based sensors have such required sensitivity.

In addition, the flexibility of modification and functionalization in graphene surfaces has opened up many possibilities for the development of tailored functional materials, so that they possess varied physicochemical properties to be employed as gas adsorbent, gas sensors, or some other electrochemical devices. Liscio et al. [38], with graphene sheets, prepared organic electronic devices, whose performances and characteristics can be controlled in a simple way by changing the concentration of the GO solution employed during the spin coating stage. In the work by Esfandiar et al. [39], the TiO_2_/graphene oxide sheet was employed to study its sensing property to H_2_, and they found that high sensitivity (92%) and fast response (no more than 20 s) could be obtained at 180 °C when the concentration of hydrogen reaches 500 ppm. In another research [40], it was demonstrated that SnO_2_ nanorods arrays on graphene sheets enhanced sensitivity to H_2_S, which is mainly attributed to the outstanding electronic characteristics of graphene and the synergism of the large surface area of SnO_2_ nanorods arrays. Based on the first principles theory, Congcong et al. [41] investigated the sensing performance of N-doped graphene to CO and put forward that such material can selectively detect CO in an environment with O_2_ and NO.

In terms of the application of graphene-based materials for gas sensors, its fundamental principle is based on the conductivity change after the adsorption of gas molecules [42]. In detail, the sorption reactions with varied gas molecules occur at the surface of graphene, where the adsorbed molecules act as donors or acceptors, leading to the transformation of carrier concentration in graphene [43] and, further, the conductivity change of graphene-based sensors. Moreover, some interesting merits of graphene-based materials can help enhance their sensitivity to varied gas molecules. For instance, Sundaram et al. [44] synthesized Pd nanoparticles modified graphene using chemical electrodeposition and found that its response to H_2_ significantly improved compared with the unmodified one, as Pd has a good affinity toward H_2_ detection. In the research by Fowler et al. [43], the graphene was chemically doped by holes to study its response to NO_2_ and NH_3_. Results showed that NO_2_ receives electrons from graphene, while NH_3_ donates electrons to graphene, resulting in different sensing mechanisms of these two gases. That is, holes induced conduction and electrons induced conduction for NO_2_ and NH_3_, respectively.

Therefore, the nature physical property of a high specific surface area allows graphene to be prepared as gas sensors for detecting individual gaseous chemicals. More importantly, the chemical modification by functional groups, metal or nonmetal atoms, as well as nanoparticle decoration can enhance the sensitivity and selectivity of graphene-based sensors to one kind of gas in the environment with mixed gases, and this improves its availability for gas sensing in related industrial applications.

## 3. Application of Graphene-Based Sensors

In view of excellent physicochemical properties of graphene-based materials discussed above, their application for preparing sensing devices have never failed to be emphasized, without the exception of fabricating related sensors employed in electrical engineering to detect SF_6_ decomposed gases. Much research has been conducted both theoretically and experimentally to investigate the adsorption mechanism and response characteristics. This section is divided into two parts to separately summarize theoretical calculations and experimental analyses.

### 3.1. Theoretical Calculations about Graphene-Based Sensors

**Figure 1:**
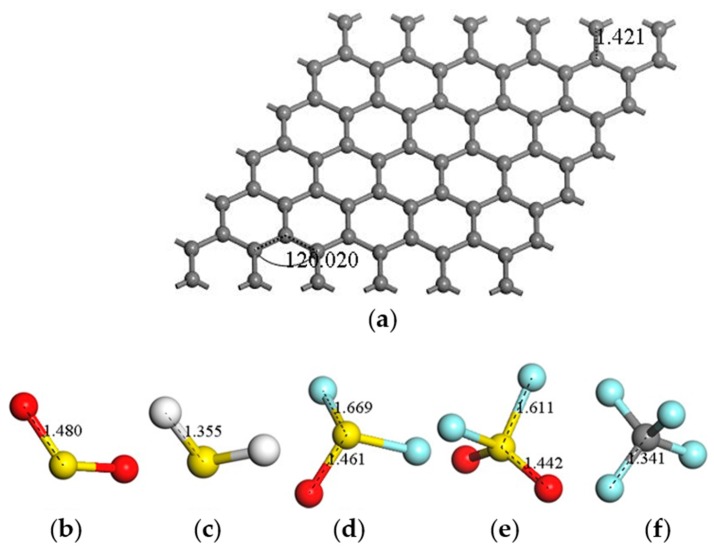
Geometric structures after optimization: (**a**) intrinsic grapheme; (**b**) SO_2_ molecule; (**c**) H_2_S molecule; (**d**) SOF_2_ molecule; (**e**) SO_2_F_2_ molecule; and (**f**) CF_4_ molecule (distances in Å).

As a complete modeling and simulation software, a materials studio was designed to help predict and understand the relationship of a material’s atomic and molecular structure with its properties and behavior [45,46]. Here it can be adopted to calculate and analyze the adsorption process between graphene-based material and SF_6_ decomposed gases. Based on first principles theory, relevant adsorbing parameters including total adsorption energies (*E*_ad_), charge transfer (*Q*_t_) value, interaction distances (*D*), energy gap (*E*_g_), and total or local density of state [45,47] can be obtained, which helps explain the adsorbing reactions. More accurately, the facts of whether the sorption can occur spontaneously or not, whether the conductivity of the material after adsorption will go up or down, whether the reaction between adsorbent and gas molecules can be deemed as physisorption or chemisorption can be known [48,49]. First, individual gas molecule models, including SO_2_F_2_, SOF_2_, H_2_S, CF_4_, and SO_2_, and the graphene model should be set up and then optimized to obtain their most stable geometric structures. Then, targeted gas molecules enable us to approach the surface of graphene so as to proceed with the adsorption process. After optimizing whole system, the related adsorbing configurations and parameters mentioned above can be gained. One thing should be mentioned that the adsorbing energy of each system could not be obtained directly. Instead, it is calculated by the following equation:

(1) $$E_{\mathrm{ad}}=E_{\mathrm{graphene}/\mathrm{molecule}}-E_{\mathrm{graphene}}-E_{\mathrm{molecule}}$$

where the *E*_graphene/molecule_, *E*_graphene_, and *E*_molecule_, respectively, represent the systemic energy after reaction, and the total energies of the graphene and the gas molecule before the reaction. A negative *E*_ad_ indicates spontaneity of the corresponding reaction. Similarly, *E*_g_ of each reaction is calculated as

(2) $$E_{g}=\left|E_{\mathrm{LUMO}}-E_{\mathrm{HOMO}}\right|\times 27.212\;\mathrm{eV}$$

where the *E*_LUMO_ and *E*_HOMO_ represent the Lowest Unoccupied Molecular Orbital and the Highest Occupied Molecular Orbital, respectively. Figure 1 shows the geometric structure of graphene and the molecule models of SF_6_ decomposed gases, which are all geometrically optimized.

In terms of theoretical calculations, plenty of research has been conducted for the purpose of discovering a novel kind of graphene-supported material that has a good gas response to SF_6_ decompositions. In the theoretical research [50], adsorption of H_2_S on intrinsic graphene is proven to be quite weak as a consequence of the small binding energy, the large bonding length, and the small net charge transfer; while for Pt-doped graphene, it has the ability to adsorb up to seven H_2_S molecules on its surface according to larger binding energy and a shorter Pt H_2_S bonding distance. Such results provide powerful proofs for the conclusion that metal-doped graphene has better gas response to gaseous chemicals analyzed before. In fact, Pt that naturally has a strong catalytic activity undoubtedly helps its doped graphene with good gas sorption [51] if it acts as an additive modifier on the surface of the graphene.

Berahman et al. [52] designed a thin film sensor-based graphene for H_2_S sensing, and they found that, compared with the film sensors prepared by intrinsic graphene, those decorated with copper (Cu) manifested better sensing behavior. Moreover, the adsorbing ability of Cu-graphene to H_2_S depends on the concentration of decorated Cu. Too much or too little Cu concentration would deteriorate the gas response of graphene-based sensors to H_2_S. Another interesting phenomenon is that the Cu decoration perfectly changes the type of the semiconductor, i.e., the intrinsic graphene-based sensors present a p-type semiconductor, while the dopant of Cu present an n-type one. Generally speaking, the doping of metal that has redundant electrons enables electrons to pad the holes and form a duplet. Once all the holes are full of electrons and certain free electrons still exist, it shows an n-type conductor; conversely, if the holes fail to be filled, it remains a p-type conductor. It is no wonder that, in this study, the Cu atom in a proper concentration provide enough electrons, leading to the transformation of the conductor type. However, too little Cu only reduces the concentration of carriers, lowering the conductivity of graphene-based sensors, while too much Cu improves the number of electrons, enhancing dramatically the conductivity of the prepared sensors. Such changes are, on the contrary, detrimental to the gas response as the conductivity changes inconspicuously after the gas adsorbing reactions. That is, they present an insensitivity to their environment. However, it is not contradictive to our previous analysis that the modification of metal can enhance sensing performance. If the dopant concentration is proper, the modified complex presents a further improved response to the target gas.

**Figure 2:**
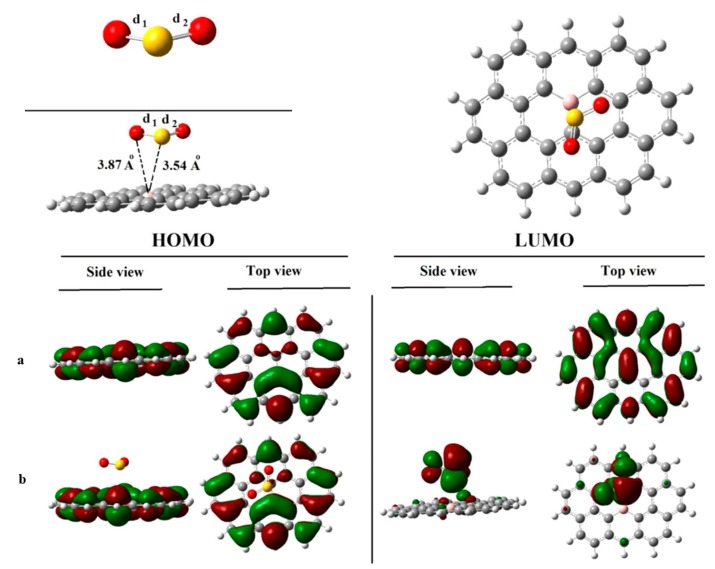
Adsorption configuration and orbital distributions for SO_2_ adsorbed on B-graphene. (**a**) isolated B-graphene; (**b**) B-graphene/SO_2_. Reproduced with permission from [55]. Copyright 2016 Journal of Solid State Chemistry.

Based on first principles theory, adsorption of SO_2_ onto light metal-doped (Li and Al) graphene oxide (GO) was investigated by Chi Chen et al. [53], and they found that the decoration of Li or Al was the main reason for the enhanced SO_2_ adsorption performance, with a binding energy of up to 1.08 eV, as the light metals that stably anchor on the GO via hydroxyl and epoxy groups provide many active adsorption sites for gas molecules. Rad et al. investigated the SO_2_ adsorption on pristine graphene, N-doped graphene [54], and B-doped graphene [55] (seen in Figure 2), and they found that the most negative *E*_ad_ comes to the adsorption when N-graphene acts as adsorbent (−19.6/mol), followed by B-graphene (−11.2 kJ/mol) and finally pristine graphene (−9.1 kJ/mol), which suggests that N-doped graphene has the best adsorbing ability to SO_2_. B-graphene shows as an n-type semiconductor because of a highly negative charge of C-atoms neighboring the boron dopant. When the SO_2_ molecule is adsorbed on the B-graphene surface, the energy gap reduces by 0.60 eV from 2.90 eV for insolated B-graphene, implying a sharp increase for conductivity of the B-graphene-based sensor. Even so, the adsorption of SO_2_ on B-graphene or intrinsic is physisorption, on N-graphene is chemisorption, as their different adsorbing cases in these two systems. The inter-molecule bond of SO_2_ witnesses little change after adsorption in SO_2_/B-graphene system due to weak sorption; while deforms destructively in SO_2_/N-graphene system because of their strong binding force.

In Figure 2, where the adsorption of SO_2_ on B-doped graphene and related orbital distributions are shown, one can find that the adsorbent-adsorbate distance is 3.54 Å, a relatively long distance manifesting their weak binding force. While the *d*_1_ (1.467 Å) and *d*_2_ (1.467 Å) present no significant change after adsorption compared with that before reaction (1.480 Å, seen in Figure 1). In terms of HOMO-LUMO distributions for the SO_2_ adsorption system, an insignificant change is presented in the HOMO distribution but LUMO localizes at the adsorbate significantly. These changes lead to a remarkable decrease in the above-mentioned *E*_g_.

**Figure 3:**
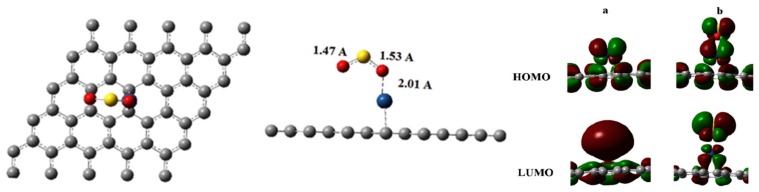
Adsorption configuration and orbital distributions for SO_2_ adsorbed on Pt-graphene. (**a**) isolated Pt-graphene; (**b**) Pt-graphene/SO_2_. Reproduced with permission from [56]. Copyright 2016 Vacuum.

In another work of Rad et al. [56], Pt-decorated graphene was employed to investigate its adsorption property to SO_2_, and found that this material exhibited good sensitivity to the analyte. The adsorbing configuration along with the orbital contributions are shown in Figure 3. It can be seen that the adsorbing distance is 2.01 Å, and the inter-molecules of SO_2_ have deformed slightly, with two S–O bonds presented to be 1.47 Å and 1.53 Å, respectively. Taking frontier molecule orbitals into consideration, one can observe that, in comparison with the bare Pt-doped graphene, notable changes are not limited to their density distributions, but also their energies. A slight increase for *E*_HOMO_ and a much more pronounced decrease *E*_LUMO_ finally decline the energy gap to 1.82 eV from 2.43 eV, which simultaneously leads to a large number of charge transfers. Through calculations, the *Q*_t_ for −0.13 e and the energy gap for 0.61 eV were obtained. The negative *Q*_t_ demonstrated that the electrons transfer from the graphene to the SO_2_ molecule, giving rise to Pt-graphene, an n-type semiconductor. A relatively high *E*_g_ guarantees the visible conductivity change of these material-based sensors, so that Pt-decorated graphene is a promising material to be prepared as gas sensors for the detection of SO_2_.

**Figure 4:**
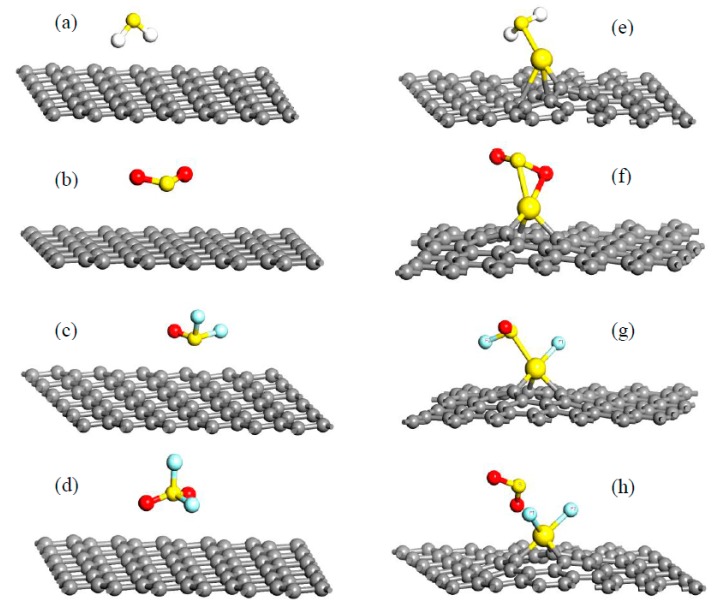
Optimized configurations for H_2_S, SO_2_, SOF_2_, and SO_2_F_2_ adsorbed on pristine graphene (**a**–**d**); optimized configurations for H_2_S, SO_2_, SOF_2_, and SO_2_F_2_ adsorbed on Au-graphene (**e**–**h**). Reproduced with permission from [57]. Copyright 2016 Applied surface science.

**Figure 5:**
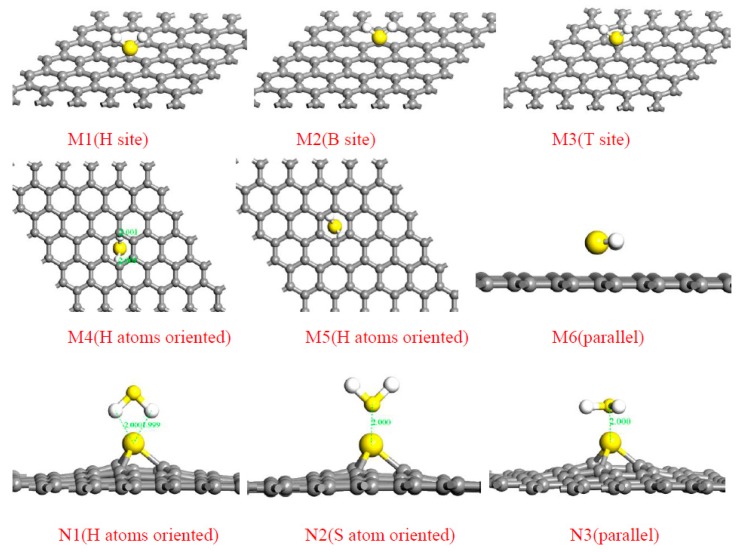
Adsorption configurations of H_2_S on pristine (M1~M6) and Au graphene surfaces (N1~N3). Reproduced with permission from [57]. Copyright 2016 Applied surface science.

Upon the work by Zhang et al. [57], they theoretically investigated the adsorption of four sorts of SF_6_ decomposed gases, SO_2_F_2_, SOF_2_, H_2_S, and SO_2_, on pristine graphene and Au-doped graphene as shown in Figure 4. All the configurations present the lowest energies among various adsorption sites with gas molecules, where the adsorptions on intrinsic graphene are shown on the left, while the adsorptions on the Au-doped one are on the right. It can be observed that, after the modification of Au on the surface of the graphene, its sorption to these gases appreciably improves. The new bonds are formed between Au and some atoms in gas molecules, and in the SO_2_F_2_ adsorbing system, two F atoms even break their bonds with the S atom and link with Au, which contributes to the strong binding forces between Au-graphene and the gas molecules. Even so, different adsorbing sites for the same gas molecule have different adsorption configurations, and related adsorbing parameters change a lot. Taking H_2_S for example, six adsorption sites for intrinsic graphene and three for Au-doped graphene were studied in this research, with corresponding adsorbing configurations and *E*_ad_ presented in Figure 5 and Table 1, respectively.

It can be seen that, although all of the *E*_ad_ are negative (demonstrating the spontaneities of these reactions), the M5 site for H_2_S adsorbed on intrinsic graphene is the most negative (−0.617 eV), indicating that the adsorption in this site occurs most easily; upon Au-graphene, the reaction for H_2_S is the most likely to take place when the S atom is oriented to the Au-graphene (N_2_ site, −0.9 eV). In terms of the other gases, the lowest *E*_ad_ among several adsorbing sites are −0.467 eV for SOF_2_/graphene with the B site, −0.961 eV for SOF_2_/Au-graphene with 2F- and S-oriented site, −0.472 eV for SO_2_F_2_/graphene with 2O- and F-oriented site, −1.790 eV for SO_2_F_2_/Au-graphene with 2F- and O-oriented site, −0.302 eV for SO_2_/graphene with the T site, and −0.587 eV for SO_2_/Au-graphene with O-oriented site, respectively. These results reveal that a much stronger adsorption reaction happens in the Au-graphene system than that of the intrinsic one. Moreover, the adsorptions between the Au-doped graphene and gas molecules are proven to be chemisorption since their *E*_ad_ are far negative than the critical value (−0.8 eV) of the chemical adsorption reported elsewhere [58]; yet for the adsorptions between intrinsic graphene and gas molecules show physisorption due to their weak sorption that are mainly dependent on Van der Waals force. This study systematically analyzed the influence of Au doping and the adsorption sites on the adsorbing ability of graphene to SF_6_ decomposition gases, coming up with a new method to research the adsorption process and a new type of possibly workable sensing material.

**Figure 6:**
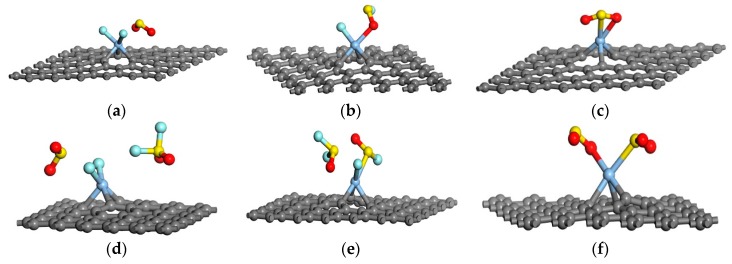
Most stable configurations of gas molecules adsorbed on the Ag-graphene (**a**) SO_2_F_2_; (**b**) SOF_2_; (**c**) SO_2_; (**d**) double SO_2_F_2_; (**e**) double SOF_2_; (**f**) double SO_2_. Reproduced with permission from reference [59]. Copyright 2016 Sensors.

In other representative research [59] based on density functional theory, the model of Ag-doped graphene, SO_2_F_2_, SOF_2_, and SO_2_ were set up, to study the adsorption behaviors and gas response of Ag modified graphene to these gases. To attain a better understanding of these adsorbing reactions and adsorbing capacity of Ag-doped complex to an individual gas, both single gas molecules and double ones were investigated in this research, with optimized geometrical structures shown in Figure 6 and related adsorbing parameters in Table 2.

In terms of one gas molecule adsorption, it can be seen that new bonds are formed in every system between certain atoms of gas molecules and Au atom on the surface of graphene, suggesting the strong binding forces of Ag-graphene to SF_6_ decompositions. Among three systems, SO_2_F_2_ shows the most active effect to Ag-doped complex with the lowest adsorption energy (−1.448 eV) and the shortest adsorption distance (2.013 Å), which means that Ag-doped graphene sensors have the best sensitivity to SO_2_F_2_ compared with the other two types of gases. Furthermore, all negative *Q*_t_ values indicate that the electrons transfer from the Ag-graphene to the gas molecules; the largest number of charges that transfer from the adsorbent to the SO_2_F_2_ molecule further verifies their strong reaction, proving that SO_2_F_2_ can be easily adsorbed on the Ag-doped complex.

Taking two gas molecules’ adsorption into consideration and comparing their adsorbing parameters with those of single gas molecule adsorption systems, it can be concluded that, for doubled SO_2_F_2_ and SOF_2_ molecules adsorption, the processes appear similar to single gas adsorption, namely that only one in two molecules interacts with Ag-graphene and adsorbed on its surface, and the other one have little interaction with it. While for double SO_2_ molecule systems, the adsorption seems to be much stronger as the *E*_ad_ become more negative and the *Q*_t_ doubled. Such conditions imply that Ag-graphene has an adsorbing capacity to SO_2_ that is superior to SO_2_F_2_ and SOF_2_. The adsorption to double SO_2_ molecules can be regarded as chemisorption, and that to SO_2_F_2_ or SOF_2_, physisorption. Even so, the *E*_ad_ and *D* in the SO_2_F_2_ system still indicate their relatively strong reaction with Ag-graphene.

Based on further studies on frontier molecular orbital theory, the HOMO, LUMO, and energy gap (*E*_g_) about different systems can be obtained to clearly understand the influence of the adsorbing gas molecules on the conductivity of Ag-graphene prepared sensors. Through analyses, it can be obtained that *E*_g_ of the SO_2_F_2_/Ag-graphene and Ag-graphene/SO_2_ systems increases sharply when the number of adsorbed gas molecules increases from 1 to 2, revealing a decreased conductivity of the graphene sensors; as for the Ag-graphene/SOF_2_ system, the number of adsorbed gas molecules may have little impact on the conductivity of graphene sensors. Overall, it was eventually concluded that the sensitivity of Ag-graphene to diverse gases agrees with the following order: SO_2_F_2_ > SO_2_ > SOF_2_, since the energy gap has little change with respect to the SOF_2_/Ag-graphene system.

### 3.2. Experimental Analyses about Graphene-Based Sensors

In terms of experiments, it also has been identified that graphene-based materials such as GO and graphene composites have a good adsorption effect and sensitivity to SF_6_ decomposition components. The natural property of GO, which owns abundant functional groups including carboxyl, hydroxyl, and epoxy groups that are all acted as energetic sites, endows its improved capacity for gas sorption, especially chemical adsorption for polar molecules such as H_2_S and SO_2_.

Seredych et al. [60,61,62,63,64] synthesized zirconium hydroxide/graphene composites, with the content of the graphene ranging from 5% to 50%, to study the adsorption of SO_2_ and H_2_S at room temperature and found that new forming basic sites and high porosity due to the reactions between zirconium hydroxide units and the oxygen groups on the graphene layers contribute to the intensive sorption for these two categories of gases. Moreover, during H_2_S adsorption, hydrogen sulfide may react with epoxy and carboxylic groups on the GO layers and further form related thio-derivatives. There is an indication that a small quantity of SO_2_ is generated on the surface of the adsorbent after H_2_S adsorption, but in most cases H_2_S is oxidized to element sulfur. In terms of SO_2_ adsorption, the catalyzed oxidation process during the peculiar physical and chemical sorption of sulfur dioxide facilitates its transformation to sulfates. Considering the superior adsorption effects of graphene composites on SO_2_ and H_2_S, it should be noted that graphene composites are promising materials for the diagnoses of insulation state.

Babu et al. [65] prepared graphene oxide (GO) to perform a gas sensing experiment on SO_2_ and found that its adsorption ability varies with the activation temperature. To prepare GO, the graphite oxide prepared by oxidation of graphite was exfoliated via a combination of ultrasound sonication and various freeze–thaw cycles. Moreover, the treatment for GO with a N_2_ plasma for 60 min was performed in order to estimate the influence of nitrogen-containing functional groups on the surface of graphene on gas sorption of SO_2_, which successfully introduces a considerable amount of N-containing functional groups because of a wide range of originally existed oxygen functional groups on GO that can contribute to the modification of N-containing groups. Through N_2_ adsorption isotherms that are used to measure the porosity and specific surface area of graphene, its surface area of after N_2_ plasma treatment was determined to be ~264 m^2^/g, slightly lower than the prepared GO (268 m^2^/g). The adsorptions were performed at different temperatures in order to study its influence on the adsorption process of SO_2_ on GO. Near ambient pressure, the adsorption abilities of GO to SO_2_ are 156 mg/g (15 °C), 153 mg/g (25 °C), and 135 mg/g (35 °C), respectively. Given the decreased adsorbing ability with the increasing temperature, the predominate adsorption of SO_2_ on GO gives rise to physisorption, which depends on a relatively strong van der Waals interaction due to the large dipole moment of SO_2_. Upon N_2_ plasma treatment on GO, a certain amount of N functional groups for ~3.5 atom % indeed is gained; however, it fails to noticeably facilitate the SO_2_ adsorption on treated GO.

**Figure 7:**
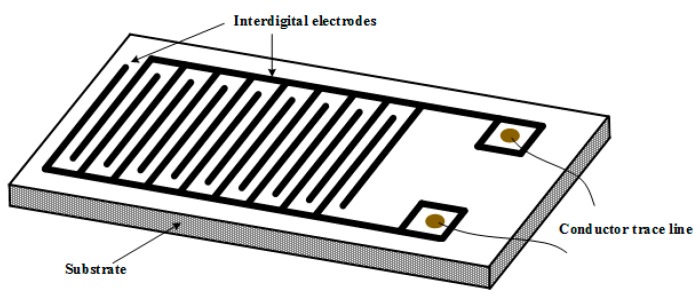
Geometrical morphology of film sensor.

Yu lei et al. [66] introduced a kind of film substrate, as shown in Figure 7, to study its sensing property with respect to one of the SF_6_ decomposition components, H_2_S, via an electrochemical workstation. The sensors were prepared by repeatedly dropping the graphene suspension onto the film with concomitant drying treatment, for the sake of obtaining a dense, uniform, and smooth graphene film on the substrate. Afterward, the sensor was exposed to environments in a sealed steal tank with different concentrates of H_2_S to determine its change in resistance.

The response of sensors is characterized as the percentage of the resistance change due to the exposure to a gas containing H_2_S against the resistance measured without the presence of sulfuretted hydrogen, expressed as [67]

(3) $$S=\frac{R-R_{0}}{R_{0}}\times 100\%$$

where *R* represents the real-time resistance of the sensors immersed in H_2_S, and *R*_0_ represents resistance measured in the initial vacuum environment. In this study, three concentrations, namely 300 ppm, 50 ppm, and 100 ppm, of H_2_S were detected to obtain the sensitivity–time curves for further analysis, expressed in Figure 6.

**Figure 8:**
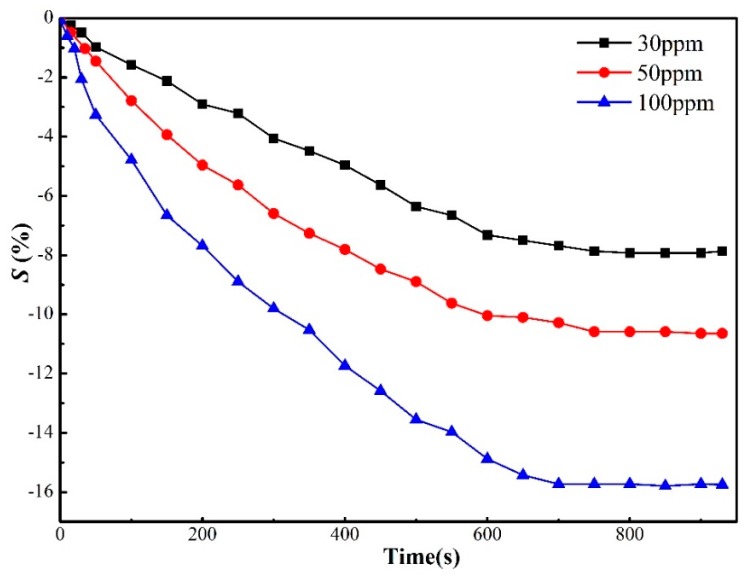
Response of graphene-based sensors to varied concentrates of H_2_S. Reproduced with permission from [66]. Copyright 2015 International Conference on High Voltage Engineering and Application.

From Figure 8, it can be seen that (i) the resistance of sensors reduces when they are immersed into the H_2_S environment; (ii) their sensitivities to H_2_S reach as high as −7.93%, −10.63%, and −15.78%, respectively, despite their poor performances in terms of long response time to H_2_S (about 10 min); (iii) the higher the concentration of H_2_S is, the higher the sensitivity of graphene-based sensors is. Through detection of H_2_S by graphene-based sensors, it can be proved that graphene-based sensors have a relatively high sensitivity to H_2_S, and the concentration of such decomposition can be approximately estimated.

In [68], Au-doped graphene was synthesized through layer-by-layer depositing graphene oxide to investigate H_2_S and SOF_2_ adsorption performances. Two concentrations of each gas (50 ppm, 100 ppm) are taken into consideration, with the sensitivity–time curves obtained under ambient condition. For H_2_S adsorption, an increased resistance of 28.15% and 18.73% are obtained for 100 ppm and 50 ppm, respectively; while upon SOF_2_ adsorption, 100 ppm and 50 ppm result in 23.83% and 15.36% decrease in resistance, respectively. These results manifest that Au-graphene can realize the selectively detection for SOF_2_ and H_2_S. Moreover, the probable concentration of individual gas can be estimated, given the dependence between resistance change and gas concentration. In comparison with the above sensitivity of graphene to H_2_S, Au-modified graphene obviously presents superior sensing behavior, leading to a better response.

**Figure 9:**
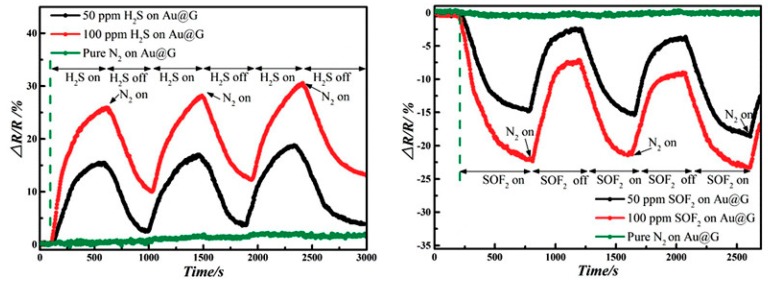
Repeated gas pulses effect of H_2_S and SOF_2_ on Au-graphene. Reproduced with permission from [68]. Copyright 2015 Advanced Science.

To investigate the repeatability of such material, the recover lines that used to explore its recovery property are obtained by injecting N_2_, as shown in Figure 9. After injection of N_2_, the existed target gas is expelled until its inexistence and subsequently begins the nest round of detection. From Figure 9, it can be observed that the sensors present a good response in the second or third round detecting test, regardless of sensitivity or response time, proving its excellent repeatability for detection of H_2_S and SOF_2_. Overall, it can be concluded that Au-graphene is a promising sensing material for detecting H_2_S and SOF_2_ according to its remarkable sensitivity, selectivity, and reusability. It is hoped that Au-doped graphene sensors can selectively detect these two gases in GIS.

## 4. Conclusions and Perspectives

In this paper, the main approaches for the synthesis of graphene, its composites, and its based materials have been summarized; simultaneously, the application of such materials in electrical engineering for the detection of SF_6_ decomposition components in order to estimate the insulation state of the power system has also been reviewed.

The technique of exfoliation, showing good promise to industrially produce graphene, TCVD, which can reproduce graphene on a centimeter scale substrate, and PECVD, which exhibits a fine feasibility to synthesize graphene on any substrate together, constitute the three main synthesis means for graphene preparation.

Based on previously theoretical and experimental researches, it can be confirmed that graphene-based materials might be a novel category of sensors because of their exceptional properties such as large specific surface area, high sensitivity, and selectivity to gases. Their outstanding sensing performances enable SF_6_ decomposed products for use in power systems, which, to a certain degree, broadens their applications, contributing to the further exploitation of their properties.

However, essential work focused on the effective preparation of graphene and relevant experiments aimed at repeatedly verifying the feasibility of graphene-based sensors should be conducted in order to realize its application on the diagnosis of GIS. First, in terms of synthesizing graphene, the inadequate ability for controlling the layer of graphene and realizing large-scale production ought to be solved. Besides, though the theoretical results prove their suitability for SF_6_ decomposition component detection, more high-accuracy experiments that lower the minimum response concentration as much as possible to meet industrial demands are still required. Not only do repeatable experiments need to be performed, but experiments about gas response to SO_2_F_2_ and SOF_2_ also need to be implemented, improving the probability of comprehensively understanding this issue.

For all of these efforts, the ultimate aim is to find a series of graphene-based sensors in which each sensor is quite sensitivity to a specific gas. It is expected that, if the sensor array prepared by graphene-based materials applied for SF_6_ decomposition detection can be built up, detection precision and accuracy can be greatly developed. That is, the individual gases of SF_6_ decomposed products could be qualitatively and quantitatively detected and, with a short response time, a high sensitivity, environmental friendliness, and chemical stability can to a large extent substitute current electrical devices.

## Figures and Tables

**Table 1:** Adsorption energies of H_2_S on pristine graphene and Au-graphene. Reproduced with permission from [57]. Copyright 2016 Applied surface science.

Sites	M1	M2	M3	M4	M5	M6	N1	N2	N3
*E*_ad_ (H_2_S) (eV)	−0.308	−0.322	−0.367	−0.521	−0.617	−0.287	−0.551	−0.9	−0.543

**Table 2:** Adsorption parameters of different systems. Reproduced with permission from [59]. Copyright 2016 Sensors.

System	D (Å)	*E*_ad_ (eV)	*Q*_t_ (e)
Ag-graphene/SO_2_F_2_	2.013	−1.448	−1.084
Ag-graphene/SOF_2_	2.029	−0.678	−0.153
Ag-graphene/SO_2_	2.210	−1.075	−0.350
Ag-graphene/2SO_2_F_2_	2.004	−1.365	−1.113
Ag-graphene/2SOF_2_	2.080	−0.503	−0.203
Ag-graphene/2SO_2_	2.210	−1.465	−0.701
